# Negative feedback loop of bone resorption by NFATc1-dependent induction of *Cadm1*

**DOI:** 10.1371/journal.pone.0175632

**Published:** 2017-04-17

**Authors:** Shinya Nakamura, Takuma Koyama, Naohiro Izawa, Seitaro Nomura, Takanori Fujita, Yasunori Omata, Takashi Minami, Morio Matsumoto, Masaya Nakamura, Eriko Fujita-Jimbo, Takashi Momoi, Takeshi Miyamoto, Hiroyuki Aburatani, Sakae Tanaka

**Affiliations:** 1 Department of Orthopaedic Surgery, Faculty of Medicine, The University of Tokyo, Bunkyo-ku, Tokyo, Japan; 2 Genome Science Division, Research Center for Advanced Science and Technology (RCAST), The University of Tokyo, Tokyo, Japan; 3 Division of Phenotype Disease Analysis, Institute of Resource Development and Analysis, Kumamoto University, Kumamoto, Kumamoto, Japan; 4 Department of Orthopedic Surgery, Keio University, Tokyo, Japan; 5 Department of Pediatrics, Jichi Medical University School of Medicine, Shimotsuke, Tochigi, Japan; 6 Department of Pathophysiology, Tokyo Medical University, Shinjuku, Tokyo, Japan; Charles P. Darby Children's Research Institute, 173 Ashley Avenue, Charleston, SC 29425, UNITED STATES

## Abstract

Trimethylation of histone H3 lysine 4 and lysine 27 (H3K4me3 and H3K27me3) at gene promoter regions critically regulates gene expression. Key developmental genes tend to exhibit changes in histone modification patterns from the H3K4me3/H3K27me3 bivalent pattern to the H3K4me3 monovalent pattern. Using comprehensive chromatin immunoprecipitation followed by sequencing in bone marrow-derived macrophages (BMMs) and mature osteoclasts, we found that cell surface adhesion molecule 1 (Cadm1) is a direct target of nuclear factor of activated T cells 1 (NFATc1) and exhibits a bivalent histone pattern in BMMs and a monovalent pattern in osteoclasts. *Cadm1* expression was upregulated in BMMs by receptor activator of nuclear factor kappa B ligand (RANKL), and blocked by a calcineurin/NFATc1 inhibitor, FK506. *Cadm1*-deficient mice exhibited significantly reduced bone mass compared with wild-type mice, which was due to the increased osteoclast differentiation, survival and bone-resorbing activity in *Cadm1*-deficient osteoclasts. These results suggest that *Cadm1* is a direct target of NFATc1, which is induced by RANKL through epigenetic modification, and regulates osteoclastic bone resorption in a negative feedback manner.

## Introduction

Skeletal homeostasis is maintained in the balance between bone resorption and bone formation. Osteoclasts (OCs) are multinucleated cells derived from monocyte/macrophage-lineage precursor cells and are primarily responsible for bone resorption [1]. Receptor activator of nuclear factor kappa B ligand (RANKL), a member of the tumor necrosis factor superfamily of cytokines, is indispensable for osteoclast differentiation [2], and its deficiency abrogates osteoclastogenesis and leads to osteopetrosis [3, 4]. RANKL binds to its receptor RANK and activates a wide range of intracellular signaling cascades that activate nuclear factor of activated T cells 1 (NFATc1), a transcription factor essential for osteoclast differentiation [5–7].

Epigenetic regulation of gene expression through post-translational modification of histone proteins by methylation and/or acetylation plays an important role in various processes such as cell cycle regulation, embryonic development and cellular differentiation [8]. Trimethylation of histone H3 at lysine 4 (H3K4me3) is mainly localized at gene promoter regions and is correlated with transcriptional activation, while trimethylation of H3 lysine 27 (H3K27me3) is involved in polycomb-mediated gene repression [9, 10]. Bernstein et al. reported that changes in the histone modification status in embryonic stem cells from H3K4me3/H3K27me3 bivalent patterns to H3K4me3 monovalent patterns by H3K27 demethylation are associated with active gene expression [11]. We previously demonstrated the bivalent to monovalent change of histone methylation at the promoter region of the *NFATc1* gene during RANKL-induced osteoclastogenesis using chromatin immunoprecipitation followed by sequencing (ChIP-seq) [12]. NFATc1 is induced by RANKL stimulation downstream of calcium/calcineurin signaling [13], and plays key roles in osteoclastogenesis by regulating the expression of various osteoclast-related genes, such as *Nfatc1* itself, *Cathepsin K* (*Ctsk*) and *dendritic cell-specific transmembrane protein* (*Dc-stamp)* [14–16].

Cell adhesion molecule 1 (CADM1), also known as IGSF4 (immunoglobulin superfamily 4), NECL2 (Nectin-like molecule 2), SynCAM1 (synaptic cell adhesion molecule 1), sgIGSF (spermatogenic immunoglobulin superfamily) or TSLC1 (tumor suppressor in lung cancer 1), is a member of the immunoglobulin superfamily. A missense mutation in the *CADM1* gene was reported in some patients with autism [17–23]. CADM1 was originally identified as a tumor suppressor gene that suppressed tumor growth in nude mice and suppressed cancer metastasis by regulating cell adhesion [24]. *Cadm1* knockout (KO) mice exhibited oligoasthenoteratozoospermia and impaired social/emotional behaviors [25, 26]. However, no skeletal phenotypes in *Cadm1* KO mice have been reported.

In this study, we explored NFATc1 target genes whose histone methylation status at the promoter regions changed from bivalent to monovalent patterns.

## Materials and methods

### Reagents

Alpha-minimum essential medium (α-MEM) and fetal bovine serum (FBS) were purchased from Life Technologies (Carlsbad, CA, USA). Recombinant human M-CSF was purchased from R&D Systems (Minneapolis, MN, USA), and GST-RANKL was purchased from Oriental Yeast Co., Ltd (Shiga, Japan). Anti-trimethyl-histone H3 lysine 4 and anti-trimethyl-histone H3 lysine 27 were from Active Motif (rabbit polyclonal antibody, 39159, Carlsbad, CA, USA) and Millipore (rabbit polyclonal antibody, 07–449, Billerica, MA, USA), respectively. Anti-NFATc1 antibody detects all DNA binding domain-containing NFATc1 splicing isoforms, but not the closely related NFATc2 [27]. Anti-FAK, anti-Pyk2 and anti-Src antibodies were from Cell Signaling Technology (rabbit polyclonal antibody; Beverly, MA, USA).

### Animal models

Wild-type mice on a C57BL/6 background were purchased from Sankyo Labo Service (Tokyo, Japan). *Cadm1* KO mice on a C57BL/6 background were generated as described [25, 26]. Mice were housed in individually ventilated cages with paper bedding and provided standard rodent maintenance diet and water throughout the study. At the studies, mice were euthanized by cervical dislocation. Ethics committee Institutional Care and Use Committee (IACUC) in the University of Tokyo specifically approved this study (P14-096).

### Cell culture

Murine bone marrow cells were isolated from the femur and tibia of male mice at 7–9 weeks of age. To prepare bone marrow macrophages (BMMs), cells were cultured in α-MEM/10% FBS with 50 ng/ml M-CSF for 2 days. BMMs were further cultured in the presence of 50 ng/ml M-CSF and 25 ng/ml RANKL for 3–5 days to generate osteoclasts.

To detect actin rings, cells were incubated for 30 min with rhodamine-conjugated phalloidin solution (Molecular Probes, Inc., Eugene, OR, USA) and observed under a fluorescence microscope (BZ-8100, Keyence).

### ChIP-seq

Cells were fixed with 1% formaldehyde at room temperature and then neutralized with glycine. Samples were then sonicated and incubated with protein A/G beads that had been pre-incubated with 4–10 μg of antibody. Immunoprecipitates were washed and reverse-crosslinked, and samples were then DNA purified using a PCR purification kit (Qiagen GmbH, Hilden, Germany). DNA libraries were prepared for sequencing using the standard Illumina protocol. Purified DNA was applied for cluster generation and sequencing was performed using the cBot Cluster Generation system and Genome Analyzer IIx system (Illumina; San Diego, CA, USA), following the manufacturer’s instructions. Obtained sequences were mapped to the reference mouse genome.

### RNA-seq

Total RNA was extracted using ISOGEN (Nippon Gene, Tokyo, Japan) from BMMs or OCs following the manufacturer’s protocol. Libraries were made using TruSeq RNA Sample Preparation Kits (Illumina), according to the manufacturer’s instructions. RNA sequencing was performed using the Illumina Genome Analyzer IIx.

### Analysis of bone mineral density (BMD) and skeletal morphology

Eight-week-old male wild-type or *Cadm1* KO mice were subcutaneously injected with 10 mg/kg body weight of calcein on 4 days and 1 day before sacrifice. Hindlimbs were removed, fixed with 70% ethanol, and subjected to a dual energy x-ray absorptiometric scan analysis to measure BMD (mg/cm^2^) using the DCS-600R system (Aloka Co. Ltd, Tokyo, Japan). Hindlimbs were then undecalcified, embedded in glycol methacrylate, cut into 3 μm sections longitudinally in the proximal region of the tibia, and stained with toluidine blue O or tartrate-resistant acid phosphatase (TRAP). For TRAP staining, tissue slices were stained at pH 5.0 in the presence of L (+)-tartaric acid using naphthol AS-MX phosphate (Sigma-Aldrich) in *N*,*N*-dimethyl formamide as substrate. Histomorphometric measurement was performed in stained sections from the secondary spongiosa area, 1.05 mm from the growth plate and 0.4 mm from the end of metaphysis, using OsteoMeasure software (OsteoMetrics, Inc., Decatur, GA).

### Bone resorption assay

Osteoclasts were cultured on dentine slices. Resorption areas were visualized by staining with 1% toluidine blue and then measured under a microscope (BZ-8100).

### Survival assay

Once osteoclasts were generated, RANKL and M-CSF were removed from the culture medium (at a time defined as time 0), and cells were cultured for the time periods indicated in the text. Survival was calculated by dividing the number of morphologically intact TRAP+ multinucleated cells by those present at time 0.

### Real-time PCR analysis

Total RNA was extracted with an RNeasy mini kit (Qiagen GmbH, Hilden, Germany) and reverse transcription was performed using a Prime Script RT reagent kit (Takara Bio) according to the manufacturer’s instructions. Realtime PCR was performed with a Thermal Cycler Dice Real-Time System using SYBR Premix Ex Taq (Takara Bio). All reactions were performed in triplicate. Relative mRNA expression levels were normalized to that of mouse *β-actin*.

Primer sequences were as follows:

*β-actin* forward, 5′-TGAGAGGGAAATCGTGCGTGAC-3′;

*β-actin* reverse, 5′-AAGAAGGAAGGCTGGAAAAGAG-3′;

*Cadm1* forward, 5′-GAAGGACAGCAGGTTTCAGC-3′;

*Cadm1* reverse, 5′-TCAACTGCCGTGTCTTTCTG-3′;

*Nfatc1* forward, 5′-CAAGTCTCACCACAGGGCTCACTA-3′;

*Nfatc1* reverse, 5′-GCGTGAGAGGTTCATTCTCCAAGT-3′;

*Ctsk* forward, 5′-GGACCCATCTCTGTGTCCAT-3′;

*Ctsk* reverse, 5′-CCGAGCCAAGAGAGCATATC-3′;

*Dc-stamp* forward, 5′-TCCTCCATGAACAAACAGTTCCAA-3′;

*Dc-stamp* reverse, 5′-AGACGTGGTTTAGGAATGCAGCTC-3′;

*Acp5* forward, 5′-TTGCGACCATTGTTAGCCACATA-3′;

*Acp5* reverse, 5′-TCAGATCCATAGTGAAACCGCAAG-3′.

### Western blotting

Cells were washed with ice-cold PBS and lysed at 4°C with RIPA buffer (1% Tween20, 0.1% SDS, 150 mM NaCl, 10 mM Tris-HCl (pH 7.4), 0.25 mM phenylmethylsulfonyl fluoride, 10 μg/ml aprotinin, 10 μg/ml leupeptin, 1 mM Na_3_VO_4_, and 5 mM NaF (Sigma)). Proteins were subjected to SDS-PAGE on 7.5–15% Tris-Glycine gradient gels or 15% Tris-Glycine gels and transferred onto PVDF membranes (Millipore Corp.). After blocking, membranes were incubated with primary antibodies to FAK, Pyk2, and Src (Cell Signaling Technology) or Actin (Sigma-Aldrich), followed by HRP-conjugated goat anti-rabbit IgG (Promega). Immunoreactive bands were visualized by ECL (GE Healthcare) according to the manufacturer’s instructions. Blots were stripped by a 30-min incubation in buffer (2% SDS, 100 mM 2-mercaptoethanol, and 62.5 mM Tris-HCl, pH 6.7) at 50°C and then re-probed with other antibodies.

### Statistical analysis

Data are presented as means ± SD. Statistical analyses were performed using a two-tailed unpaired Student’s *t-*test.

## Results

### *Cadm1* exhibits changes from the H3K4me3/H3K27me3 bivalent to the H3K4me3 monovalent pattern and is an NFATc1 target in osteoclasts

We first identified genes that exhibited H3K4me3/H3K27me3 bivalent patterns at the promoter region in BMMs and H3K4me3 monovalent patterns in mature osteoclasts by comprehensive ChIP-seq analysis using anti-H3K4me3 and anti-H3K27me3 antibodies, as previously described [12, 28, 29]. We selected 49 genes that showed the change of the histone methylation pattern within 5 kb of transcriptional start sites (TSSs), which are putative promoter regions. We also performed ChIP-seq using anti-NFATc1 antibody, and found that 33 genes among the 49 genes exhibited NFATc1 binding at the promoter regions in osteoclasts. These genes included representative osteoclast-specific genes, such as *Nfatc1* and *carbonic anhydrase 2* (*Car2*) (Table 1) [14, 30].

**Table 1:** Genes whose histone modification pattern changes from bivalent to monovalent during osteoclastogenesis and with NFATc1 binding near the transcription start site.

*4930506M07Rik*	*D10Bwg1379e*	*Rab34*
*Adcy3*	*Edil3*	*Rab38*
*Arhgef12*	*Fbxo32*	*Rnd1*
*Atrnl1*	*Lgals3*	*Rps6ka2*
*Bahd1*	*Met*	*Rusc2*
*Cadm1*	*Msi2*	*Sdc1*
*Car2*	*Myo1d*	*Sh3bgrl2*
*Cd109*	*Nfatc1*	*Tmcc3*
*Cd97*	*Plxna2*	*Tspan17*
*Cdyl2*	*Plxnd1*	*Vegfa*
*Cgnl1*	*Ppap2a*	*Zfp462*

**Fig 1:**
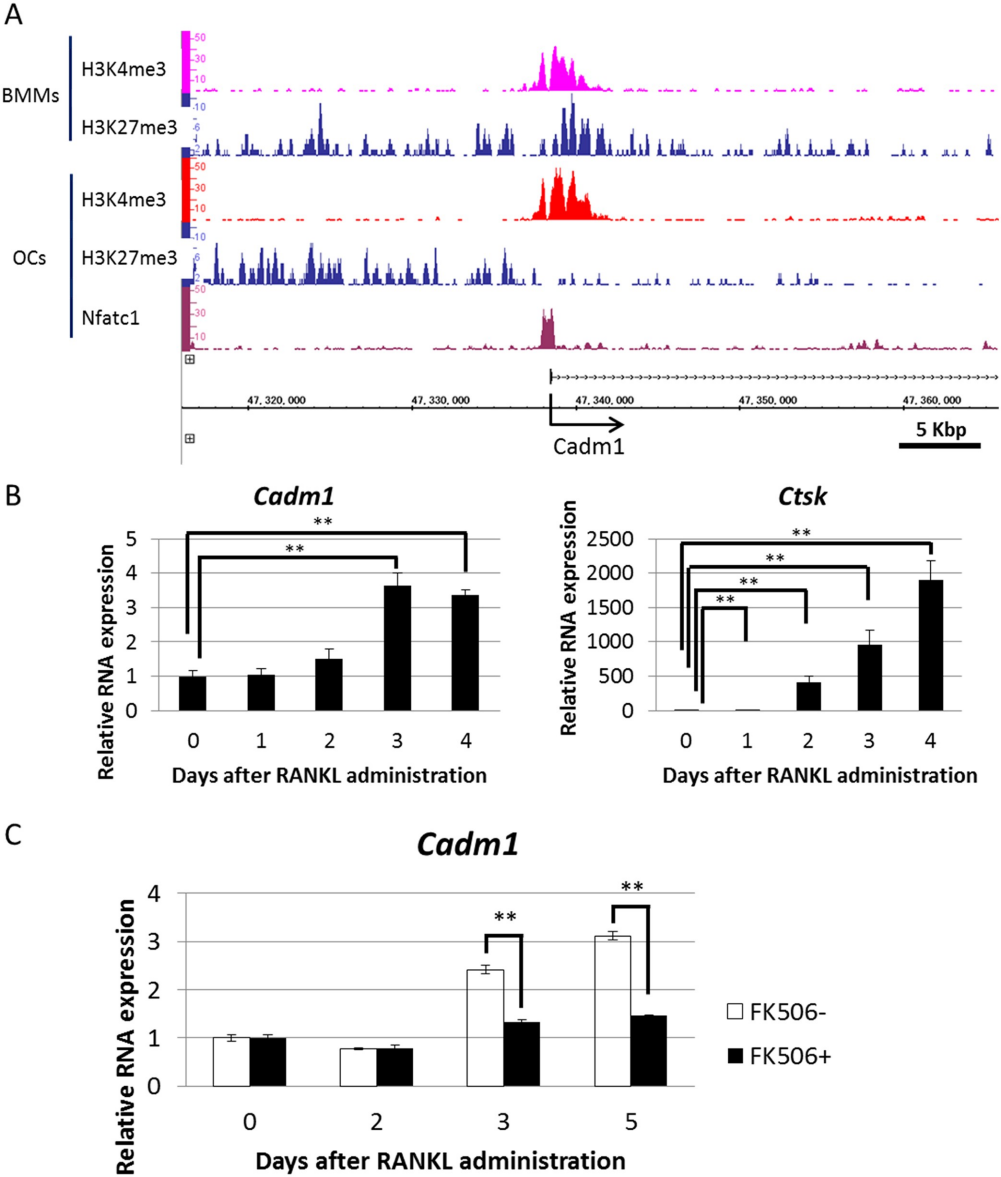
Epigenetic regulation and expression of *Cadm1* during osteoclastogenesis. (A) ChIP-seq analysis of H3K4me3, H3K27me3 and NFATc1 near the *Cadm1* transcription start site (TSS). (B and C) Expression of *Cadm1* and *Ctsk* mRNAs relative to that of *β-actin* in BMMs cultured in the presence of M-CSF (50 ng/ml) and RANKL (25 ng/ml) with or without FK506 (1 μM) for indicated periods. Data represent mean *Cadm1* or *Ctsk* expression relative to *β-actin* ± SD (*P < 0.05; *n* = 3).

Among the 33 identified genes, we focused on *Cadm1*, which encodes a cell adhesion molecule belonging to the immunoglobulin superfamily [24]. Immunoglobulin superfamily proteins played a critical role in RANKL-induced osteoclastogenesis by regulating intracellular calcium signaling [31]. In addition, members such as intercellular adhesion molecule-1 (ICAM1) played critical roles in osteoclast function [31]. Therefore, Cadm1 may regulate osteoclast differentiation and function via its immunoglobulin-like motifs. Histone modifications at the *Cadm1* promoter changed from H3K4me3/H3K27me3 bivalent patterns in BMMs to H3K4me3 monovalent patterns in response to RANKL (Fig 1A). NFATc1 binding at the *Cadm1* promoter in osteoclasts was detected by ChIP-seq (Fig 1A), confirming our selection criteria. Real-time PCR analysis revealed that *Cadm1* expression was greatly increased during RANKL-stimulated osteoclastogenesis and positively correlated with elevated expression of *Ctsk*, a marker of osteoclast differentiation (Fig 1B). RANKL-dependent *Cadm1* upregulation in osteoclasts was blocked by treatment of cells with a calcium/calcineurin signaling inhibitor, FK506, confirming that *Cadm1* is an NFATc1 target (Fig 1C).

### *Cadm1* KO mice exhibit decreased bone mass

**Fig 2:**
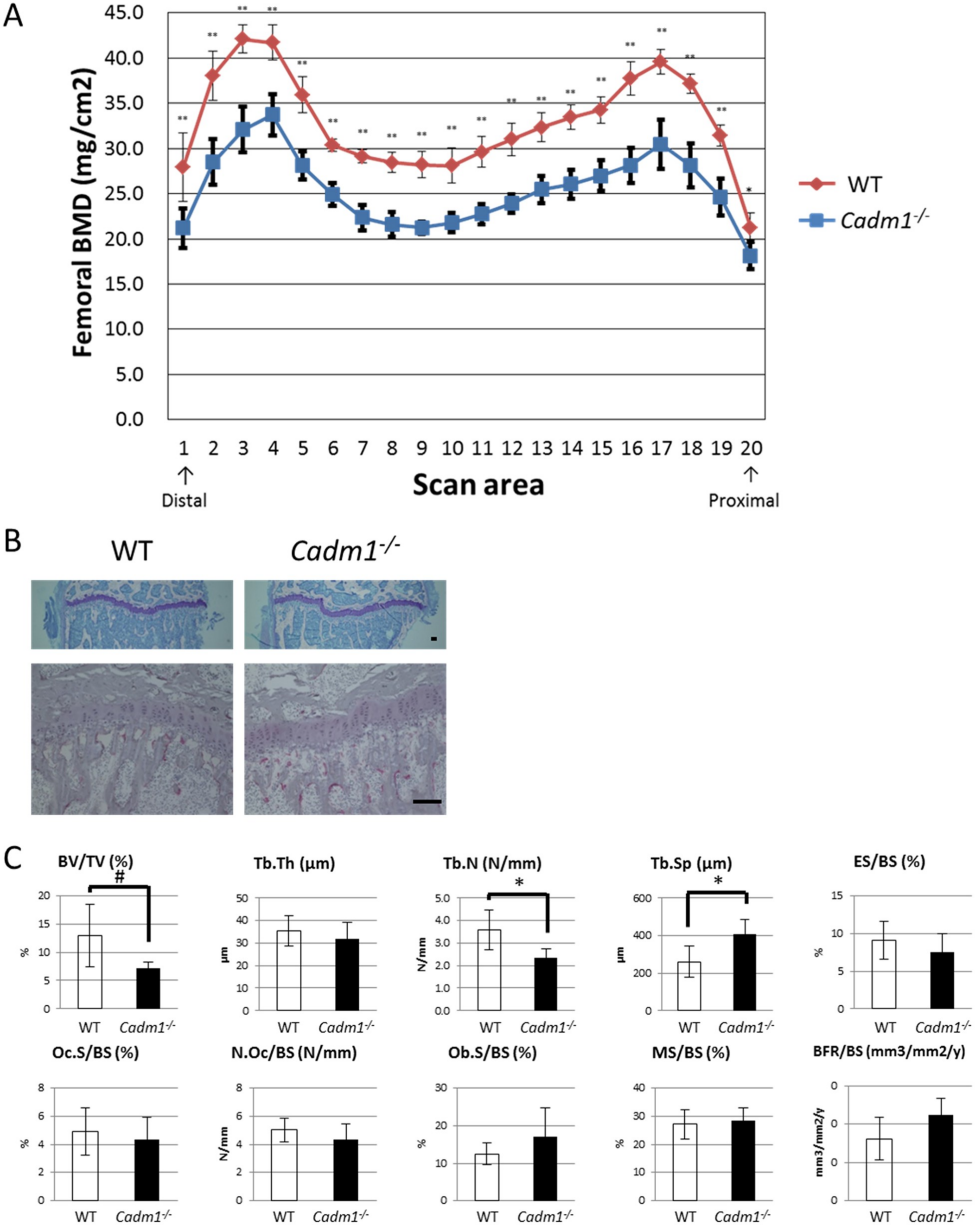
*Cadm1* KO mice exhibit decreased bone mineral density. (A) Bone mineral density (BMD) of femurs from male wild-type or *Cadm1* KO mice (*P < 0.05; **P < 0.005; *n* = 5). (B) Representative toluidine blue O staining (top) and TRAP staining (bottom) images of proximal tibias from male wild-type (left) or *Cadm1* KO (right) mice. Bars, 100 μm. (C) Bone morphometric analysis of 8-week-old wild-type or *Cadm1* KO mice. BV/TV, bone volume per total volume; Tb.Th, trabecular thickness; Tb.N, trabecular number; Tb.Sp, trabecular separation; ES/BS, eroded surface per bone surface; Oc.S/BS, osteoclast surface per bone surface; N.Oc/BS, osteoclast number per bone surface; Ob.S/BS, osteoblast surface per bone surface; MS/BS, mineralizing surface per bone surface; BFR/BS, bone formation rate per bone surface. Data represent mean value of the indicated parameter ± SD (#*P* < 0.1; *P < 0.05; *n* = 5).

We next analyzed the skeletal tissue of *Cadm1* KO mice. *Cadm1* KO mice showed a significant reduction in bone mass compared with wild-type mice (Fig 2A). Bone morphometric analysis revealed that *Cadm1* KO mice exhibited reduced BV/TV and Tb.N and elevated Tb.Sp (Fig 2B and 2C). In contrast, osteoclast parameters, such as ES/BS, Oc.S/BS and N.Oc/BS, or osteoblast parameters, such as Ob.S/BS, MS/BS and BFR, were not significantly different between wild-type and KO mice (Fig 2B and 2C).

### *Cadm1* KO osteoclasts show enhanced differentiation and bone-resorbing function

**Fig 3:**
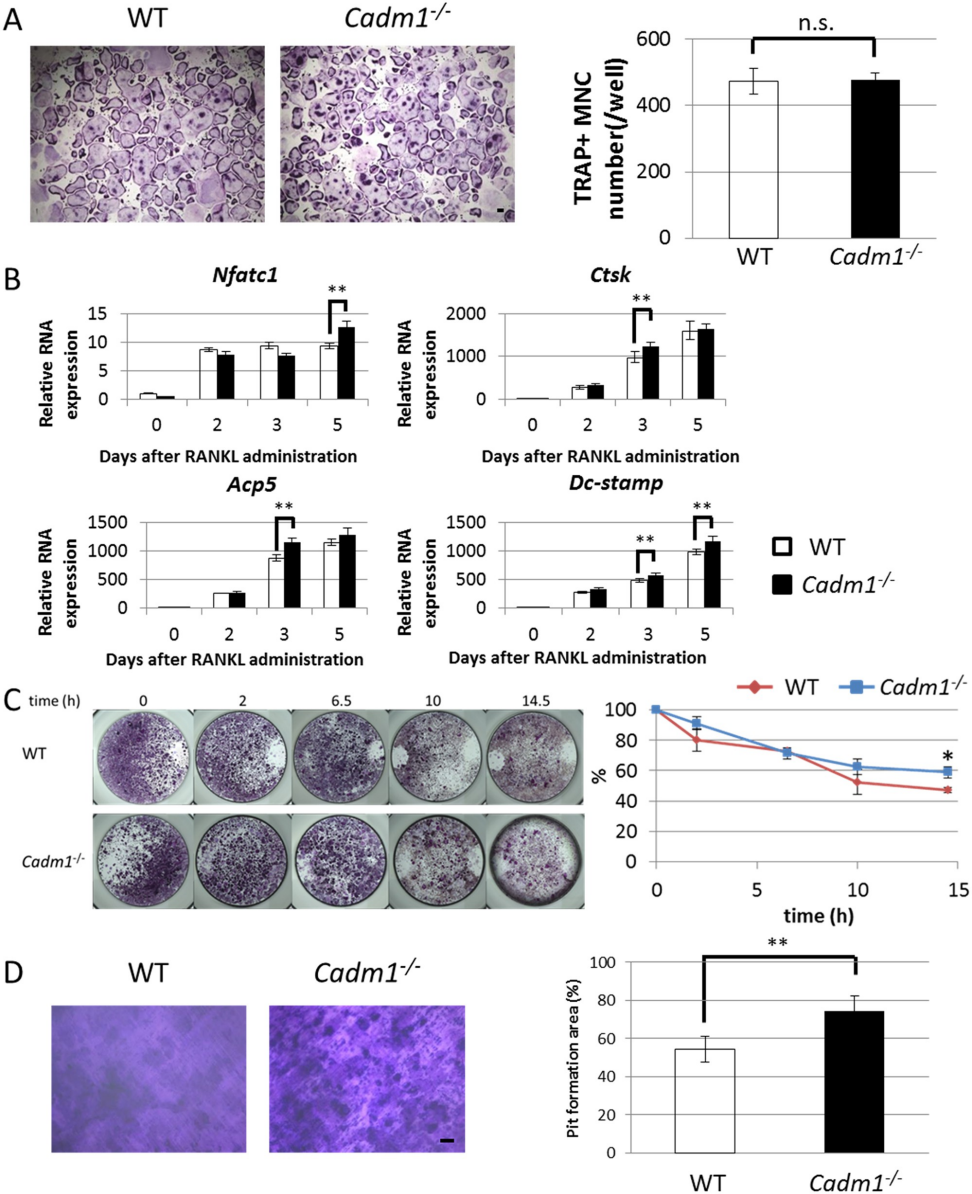
*Cadm1* loss stimulates osteoclast differentiation and enhances survival and bone-resorption. (A and B) BMMs from wild-type or *Cadm1* KO mouse were cultured in the presence of M-CSF (50 ng/ml) and RANKL (25 ng/ml) for 4 days and then stained for TRAP (A) or evaluated by real-time PCR for expression of the indicated osteoclast markers (B). TRAP+ multinucleated cells (MNCs) containing more than three nuclei were counted as osteoclasts. Data represent mean number of osteoclasts ± SD (n.s., not significant; *n* = 3). Bar, 100 μm. Data represent mean *Nfatc1*, *Cathepsin K* (*Ctsk*), *acid phosphatase 5*, *tartrate resistant* (*Acp5*) or *dendritic cell-specific transmembrane protein* (*Dc-stamp*) expression relative to that of *β-actin* ± SD (B, **P < 0.01; *n* = 6). (C) BMMs from wild-type or *Cadm1* KO mouse were cultured in the presence of M-CSF (50 ng/ml) and RANKL (25 ng/ml) for 4 days. M-CSF and RANKL were then removed from the medium, and cells were stained with TRAP at indicated times after cytokine withdrawal. Remaining TRAP+ cells were scored as surviving cells. Data represent mean number of surviving cells per well (%) ± SD (*P < 0.05; *n* = 4). (D) BMMs from wild-type or *Cadm1* KO mouse were cultured in the presence of M-CSF (50 ng/ml) and RANKL (25 ng/ml) on dentine slices for 4 days; resorption areas were visualized by toluidine blue staining (left) and the resorption area was scored. Data represent mean resorption area (%) ± SD (**P < 0.01; *n* = 6).

To analyze the possible function of Cadm1 in osteoclasts, we isolated osteoclast progenitor cells from *Cadm1* KO and wild-type mice and cultured them in the presence of M-CSF and RANKL (Fig 3). Osteoclast differentiation as analyzed by multinucleated TRAP-positive cell formation did not differ between *Cadm1* KO and wild-type cells (Fig 3A). In addition, nuclear localization of Nfatc1 was not different between *cadm1*-deficient and wild-type osteoclasts (S1 Fig). However, real-time PCR analysis indicated that expressions of osteoclast-specific genes such as *Nfatc1*, *Ctsk*, *acid phosphatase 5*, *tartrate resistant (Acp5)* and *Dc-stamp* were slightly but significantly higher in *Cadm1* KO osteoclasts as compared with wild-type cells (Fig 3B). In addition, *Cadm1* KO osteoclasts were more resistant to cytokine depletion-induced cell death as compared with wild-type osteoclasts (Fig 3C). Furthermore, bone-resorbing activity, as evaluated by pit formation on dentine slices, was significantly higher in *Cadm1* KO osteoclasts than wild-type osteoclasts (Fig 3D). Overall, these results suggest that Cadm1 negatively regulates osteoclast differentiation and function.

### *Cadm1* KO osteoclasts show enhanced expression of adhesion-related factors

**Fig 4:**
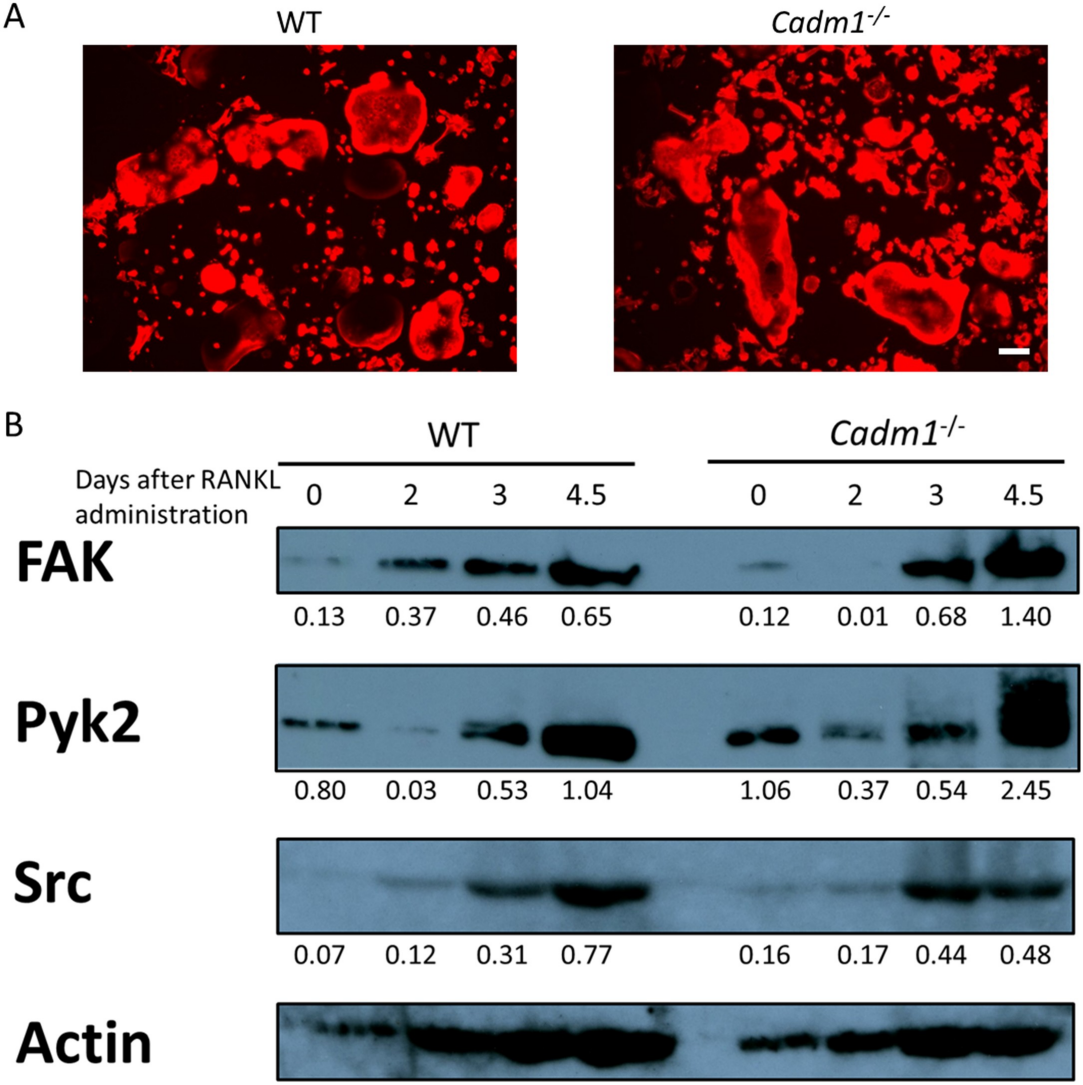
*Cadm1* ablation enhances bone-resorbing activity of osteoclasts. (A) BMMs from wild-type or *Cadm1* KO mouse were cultured in the presence of M-CSF (50 ng/ml) and RANKL (25 ng/ml) for 4 days, stained with rhodamine phalloidin to visualize actin protein, and observed under a fluorescence microscope. Bar, 100 μm. (B) Whole cell lysates from wild-type or *Cadm1* KO osteoclasts were subjected to western blotting using antibodies against FAK, Pyk2 and Src. Actin protein expression served as an internal control. Protein levels relative to actin were quantified by densitometry and are shown below.

Given that *Cadm1* KO osteoclasts exhibited elevated bone-resorbing activity (Fig 3D), we first analyzed the formation of actin rings, which are cytoskeletal structures essential for the bone-resorbing activity of osteoclasts (Fig 4A). However, our results showed that actin rings were similarly formed in *Cadm1* KO and wild-type osteoclasts (Fig 4A). We next analyzed the expression of FAK, Pyk2 and c-Src, signaling molecules implicated in cell adhesion and cytoskeletal organization of osteoclasts [32]. c-Src protein levels as assessed by western blot were equivalent between *Cadm1* KO and wild-type osteoclasts, while FAK and Pyk2 levels were elevated in *Cadm1* KO osteoclasts (Fig 4B).

## Discussion

Epigenetic modifications are critical in the organization of chromatin structures at various levels and therefore regulate gene expression [33]. Among the various epigenetic modifications, histone methylation is associated with both transcriptionally active and repressive chromatin status. The methylated sites in histones H3 or H4 are mainly located in the histone tail (H3K4, H3K9, H3K36 and H4K20) and the center of the nucleosome (H3K79) [34]. In this study, we explored NFATc1 target genes whose histone methylation status at the promoter regions changed from bivalent to monovalent patterns, and we identified *Cadm1* gene as a candidate. CADM1 is an immunoglobulin-like cell adhesion molecule that regulates intercellular attachment through homophilic interactions [20, 24]. Recent studies showed that the heterophilic interaction between CADM1 and other molecules also plays a critical role in various types of cells. For example, the attachment and functional interaction between dorsal root ganglia neurons and mast cells are mediated by heterophilic binding between mast cell CADM1 and neuronal nectin-3 [35]. The intracellular domain of CADM1 contains FERM (protein 4.1/ezrin/radixin/moesin)-binding and PDZ-binding motifs, which potentially act as a molecular scaffold to form signaling complexes [36]. Stagi et al. reported that FAK binds to CADM1 independently of PDZ domains, but requires the FERM motif, and is implicated in the morphogenetic activities of growth cones [37]. These studies suggest that CADM1 regulates cell-cell interactions and cytoskeletal organization.

Our findings showed that *Cadm1* expression was induced by RANKL in BMMs in an NFATc1-dependent manner, and histone modification at its promoter regions changed from bivalent to monovalent patterns in response to RANKL. Osteoclasts differentiated from *Cadm1* KO mouse BMMs showed higher expression of osteoclast differentiation markers such as *Nfatc1*, *Ctsk*, *Acp5* and *Dc-stamp* than wild-type osteoclasts. In addition, osteoclasts differentiated from *Cadm1* KO mouse BMMs showed enhanced bone-resorbing activity and survival. The increased bone-resorbing activity of *Cadm1* KO osteoclasts is at least partly due to the increased expression of FAK and Pyk2, which are intracellular signaling molecules that play critical roles in the cytoskeletal organization in osteoclasts [32, 38, 39]. However, the mechanisms by which Cadm1 regulates FAK and Pyk2 expression in osteoclasts remain elusive. Consistent with these *in vitro* findings, *Cadm1* KO mice exhibited reduced bone mass. However, bone resorption parameters such as ES/BS, Oc.S/BS and N.Oc/BS were not different between wild type and KO mice. The reason for this discrepancy is unclear, but it may be because the marked reduction of cancellous bone may hamper the accurate measurement of histomorphometric markers.

It is well established that immunoreceptor tyrosine-based activation motif (ITAM) signaling plays a pivotal role in osteoclast differentiation. Both DNAX activating protein of 12 kDa (DAP12) and Fc receptor common γ chain (FcRγ) contain cytoplasmic ITAM motif, and mice lacking DAP12 and FcRγ exhibit severe osteopetrosis due to the lack of osteoclasts [40, 41]. BMMs obtained from DAP12 knock out mouse macrophages undergo osteoclastogenesis in response to RANKL but fail to form actin rings, indicating the role of DAP12 in the cytoskeletal organization as well [42]. It was recently reported that sialic acid binding immunoglobulin-like lectin (Siglec)-15 is essential for the functional osteoclast differentiation. Siglec-15 is associated with DAP12 and regulates osteoclast differentiation through ITAM-mediated signaling pathways [43, 44]. Although the interaction between Cadm1 and DAP12 remains unknown, it is possible that Cadm1 negatively regulates osteoclast differentiation by antagonizing Siglec-15 and DAP12 interaction.

Several cytokines and chemokines induced by RANKL promote osteoclast differentiation, thus forming a positive feedback loop in osteoclastogenesis [45]. Conversely, negative regulators of osteoclastogenesis such as interferon regulatory factor 8 (Irf8) and Maf are downregulated by RANKL through B lymphocyte-induced maturation protein-1 (Blimp-1), which is induced by NFATc1 [45]. Here we demonstrated the possibility that the negative regulator Cadm1 is directly regulated by NFATc1 through epigenetic modifications. Our results may provide a novel mechanism for the fine-tuning of osteoclastogenesis through epigenetic and NFATc1-dependent mechanisms.

## Conclusions

Cadm1 expression is induced by changes in epigenetic modification and by NFATc1 in osteoclasts following RANKL stimulation and serves to inhibit osteclastogenesis.

## Supporting information

S1 Fig BMMs from wild-type or cadm1 KO mice were cultured in the presence of M-CSF (50 ng/ml) and RANKL (25 ng/ml) for 4 days. The cells were stained for Nfatc1 using mouse anti-NFATc1 antibody (mouse monoclonal antibody; Santa Cruz Biotechnology, Inc., Dallas, TX, USA) followed by Alexa546-conjugated goat anti-mouse IgG (Invitrogen, Carlsbad, CA, USA). DAPI (Wako Pure Chemicals Industries, Osaka, Japan) was used for a nuclear staining. Nfatc1 was visualized in red, DAPI was double-labeled in blue, and merged images were shown. Bar = 100 μm.
(TIF)

## References

[1] Boyle WJ, Simonet WS, Lacey DL. Osteoclast differentiation and activation. Nature. 2003;423(6937):337–42. 10.1038/nature01658 12748652

[2] Tanaka S. Signaling axis in osteoclast biology and therapeutic targeting in the RANKL/RANK/OPG system. Am J Nephrol. 2007;27(5):466–78. 10.1159/000106484 17652963

[3] Theill LE, Boyle WJ, Penninger JM. RANK-L and RANK: T cells, bone loss, and mammalian evolution. Annu Rev Immunol. 2002;20:795–823. 10.1146/annurev.immunol.20.100301.064753 11861618

[4] Lazner F, Gowen M, Pavasovic D, Kola I. Osteopetrosis and osteoporosis: two sides of the same coin. Hum Mol Genet. 1999;8(10):1839–46. 10469835 10.1093/hmg/8.10.1839

[5] Asagiri M, Sato K, Usami T, Ochi S, Nishina H, Yoshida H, et al Autoamplification of NFATc1 expression determines its essential role in bone homeostasis. The Journal of experimental medicine. 2005;202(9):1261–9. 10.1084/jem.20051150 16275763 PMC2213228

[6] Matsuo K, Galson DL, Zhao C, Peng L, Laplace C, Wang KZ, et al Nuclear factor of activated T-cells (NFAT) rescues osteoclastogenesis in precursors lacking c-Fos. J Biol Chem. 2004;279(25):26475–80. 10.1074/jbc.M313973200 15073183

[7] Takayanagi H, Kim S, Koga T, Nishina H, Isshiki M, Yoshida H, et al Induction and activation of the transcription factor NFATc1 (NFAT2) integrate RANKL signaling in terminal differentiation of osteoclasts. Dev Cell. 2002;3(6):889–901. 12479813 10.1016/s1534-5807(02)00369-6

[8] Goldberg AD, Allis CD, Bernstein E. Epigenetics: a landscape takes shape. Cell. 2007;128(4):635–8. 10.1016/j.cell.2007.02.006 17320500

[9] Strahl BD, Ohba R, Cook RG, Allis CD. Methylation of histone H3 at lysine 4 is highly conserved and correlates with transcriptionally active nuclei in Tetrahymena. Proc Natl Acad Sci U S A. 1999;96(26):14967–72. 10611321 10.1073/pnas.96.26.14967 PMC24756

[10] Schwartz YB, Pirrotta V. Polycomb silencing mechanisms and the management of genomic programmes. Nature reviews Genetics. 2007;8(1):9–22. 10.1038/nrg1981 17173055

[11] Bernstein BE, Mikkelsen TS, Xie X, Kamal M, Huebert DJ, Cuff J, et al A bivalent chromatin structure marks key developmental genes in embryonic stem cells. Cell. 2006;125(2):315–26. 10.1016/j.cell.2006.02.041 16630819

[12] Yasui T, Kadono Y, Nakamura M, Oshima Y, Matsumoto T, Masuda H, et al Regulation of RANKL-induced osteoclastogenesis by TGF-beta through molecular interaction between Smad3 and Traf6. J Bone Miner Res. 2011;26(7):1447–56. 10.1002/jbmr.357 21305609

[13] Hirotani H, Tuohy NA, Woo JT, Stern PH, Clipstone NA. The calcineurin/nuclear factor of activated T cells signaling pathway regulates osteoclastogenesis in RAW264.7 cells. J Biol Chem. 2004;279(14):13984–92. 10.1074/jbc.M213067200 14722106

[14] Takayanagi H. Osteoimmunology: shared mechanisms and crosstalk between the immune and bone systems. Nat Rev Immunol. 2007;7(4):292–304. 10.1038/nri2062 17380158

[15] Kim Y, Sato K, Asagiri M, Morita I, Soma K, Takayanagi H. Contribution of nuclear factor of activated T cells c1 to the transcriptional control of immunoreceptor osteoclast-associated receptor but not triggering receptor expressed by myeloid cells-2 during osteoclastogenesis. J Biol Chem. 2005;280(38):32905–13. 10.1074/jbc.M505820200 16046394

[16] Matsumoto M, Kogawa M, Wada S, Takayanagi H, Tsujimoto M, Katayama S, et al Essential role of p38 mitogen-activated protein kinase in cathepsin K gene expression during osteoclastogenesis through association of NFATc1 and PU.1. J Biol Chem. 2004;279(44):45969–79. 10.1074/jbc.M408795200 15304486

[17] Zhiling Y, Fujita E, Tanabe Y, Yamagata T, Momoi T, Momoi MY. Mutations in the gene encoding CADM1 are associated with autism spectrum disorder. Biochemical and biophysical research communications. 2008;377(3):926–9. 10.1016/j.bbrc.2008.10.107 18957284

[18] Biederer T, Stagi M. Signaling by synaptogenic molecules. Curr Opin Neurobiol. 2008;18(3):261–9. 10.1016/j.conb.2008.07.014 18725297 PMC2633430

[19] Gomyo H, Arai Y, Tanigami A, Murakami Y, Hattori M, Hosoda F, et al A 2-Mb sequence-ready contig map and a novel immunoglobulin superfamily gene IGSF4 in the LOH region of chromosome 11q23.2. Genomics. 1999;62(2):139–46. 10.1006/geno.1999.6001 10610705

[20] Kuramochi M, Fukuhara H, Nobukuni T, Kanbe T, Maruyama T, Ghosh HP, et al TSLC1 is a tumor-suppressor gene in human non-small-cell lung cancer. Nat Genet. 2001;27(4):427–30. 10.1038/86934 11279526

[21] Takai Y, Miyoshi J, Ikeda W, Ogita H. Nectins and nectin-like molecules: roles in contact inhibition of cell movement and proliferation. Nat Rev Mol Cell Biol. 2008;9(8):603–15. 10.1038/nrm2457 18648374

[22] Urase K, Soyama A, Fujita E, Momoi T. Expression of RA175 mRNA, a new member of the immunoglobulin superfamily, in developing mouse brain. Neuroreport. 2001;12(15):3217–21. 11711859 10.1097/00001756-200110290-00015

[23] Wakayama T, Ohashi K, Mizuno K, Iseki S. Cloning and characterization of a novel mouse immunoglobulin superfamily gene expressed in early spermatogenic cells. Mol Reprod Dev. 2001;60(2):158–64. 10.1002/mrd.1072 11553913

[24] Murakami S, Sakurai-Yageta M, Maruyama T, Murakami Y. Trans-homophilic interaction of CADM1 activates PI3K by forming a complex with MAGuK-family proteins MPP3 and Dlg. PLoS One. 2014;9(9):e110062 10.1371/journal.pone.0110062 24503895 PMC3913574

[25] Fujita E, Kouroku Y, Ozeki S, Tanabe Y, Toyama Y, Maekawa M, et al Oligo-astheno-teratozoospermia in mice lacking RA175/TSLC1/SynCAM/IGSF4A, a cell adhesion molecule in the immunoglobulin superfamily. Mol Cell Biol. 2006;26(2):718–26. 10.1128/MCB.26.2.718-726.2006 16382161 PMC1346906

[26] Takayanagi Y, Fujita E, Yu Z, Yamagata T, Momoi MY, Momoi T, et al Impairment of social and emotional behaviors in Cadm1-knockout mice. Biochemical and biophysical research communications. 2010;396(3):703–8. 10.1016/j.bbrc.2010.04.165 20450890

[27] Suehiro J, Kanki Y, Makihara C, Schadler K, Miura M, Manabe Y, et al Genome-wide approaches reveal functional vascular endothelial growth factor (VEGF)-inducible nuclear factor of activated T cells (NFAT) c1 binding to angiogenesis-related genes in the endothelium. The Journal of biological chemistry. 2014;289(42):29044–59. 10.1074/jbc.M114.555235 25157100 PMC4200259

[28] Nakamura H, Nakashima T, Hayashi M, Izawa N, Yasui T, Aburatani H, et al Global epigenomic analysis indicates protocadherin-7 activates osteoclastogenesis by promoting cell-cell fusion. Biochemical and biophysical research communications. 2014;455(3–4):305–11. 10.1016/j.bbrc.2014.11.009 25446128

[29] Omata Y, Nakamura S, Koyama T, Yasui T, Hirose J, Izawa N, et al Identification of Nedd9 as a TGF-beta-Smad2/3 Target Gene Involved in RANKL-Induced Osteoclastogenesis by Comprehensive Analysis. PLoS One. 2016;11(6):e0157992 10.1371/journal.pone.0157992 27336669 PMC4918979

[30] Sly WS, Hu PY. Human carbonic anhydrases and carbonic anhydrase deficiencies. Annu Rev Biochem. 1995;64:375–401. 10.1146/annurev.bi.64.070195.002111 7574487

[31] Negishi-Koga T, Gober HJ, Sumiya E, Komatsu N, Okamoto K, Sawa S, et al Immune complexes regulate bone metabolism through FcRgamma signalling. Nat Commun. 2015;6:6637 10.1038/ncomms7637 25824719

[32] Ray BJ, Thomas K, Huang CS, Gutknecht MF, Botchwey EA, Bouton AH. Regulation of osteoclast structure and function by FAK family kinases. J Leukoc Biol. 2012;92(5):1021–8. 10.1189/jlb.0512259 22941736 PMC3476245

[33] Margueron R, Trojer P, Reinberg D. The key to development: interpreting the histone code? Curr Opin Genet Dev. 2005;15(2):163–76. 10.1016/j.gde.2005.01.005 15797199

[34] Mersfelder EL, Parthun MR. The tale beyond the tail: histone core domain modifications and the regulation of chromatin structure. Nucleic Acids Res. 2006;34(9):2653–62. 10.1093/nar/gkl338 16714444 PMC1464108

[35] Furuno T, Hagiyama M, Sekimura M, Okamoto K, Suzuki R, Ito A, et al Cell adhesion molecule 1 (CADM1) on mast cells promotes interaction with dorsal root ganglion neurites by heterophilic binding to nectin-3. J Neuroimmunol. 2012;250(1–2):50–8. 10.1016/j.jneuroim.2012.05.016 22703826

[36] Yageta M, Kuramochi M, Masuda M, Fukami T, Fukuhara H, Maruyama T, et al Direct association of TSLC1 and DAL-1, two distinct tumor suppressor proteins in lung cancer. Cancer Res. 2002;62(18):5129–33. 12234973

[37] Stagi M, Fogel AI, Biederer T. SynCAM 1 participates in axo-dendritic contact assembly and shapes neuronal growth cones. Proc Natl Acad Sci U S A. 2010;107(16):7568–73. 10.1073/pnas.0911798107 20368431 PMC2867738

[38] Tanaka S, Takahashi N, Udagawa N, Murakami H, Nakamura I, Kurokawa T, et al Possible involvement of focal adhesion kinase, p125FAK, in osteoclastic bone resorption. J Cell Biochem. 1995;58(4):424–35. 10.1002/jcb.240580405 7593264

[39] Xiong WC, Feng X. PYK2 and FAK in osteoclasts. Front Biosci. 2003;8:d1219–26. 12957821 10.2741/1117

[40] Koga T, Inui M, Inoue K, Kim S, Suematsu A, Kobayashi E, et al Costimulatory signals mediated by the ITAM motif cooperate with RANKL for bone homeostasis. Nature. 2004;428(6984):758–63. 10.1038/nature02444 15085135

[41] Mocsai A, Humphrey MB, Van Ziffle JA, Hu Y, Burghardt A, Spusta SC, et al The immunomodulatory adapter proteins DAP12 and Fc receptor gamma-chain (FcRgamma) regulate development of functional osteoclasts through the Syk tyrosine kinase. Proc Natl Acad Sci U S A. 2004;101(16):6158–63. 10.1073/pnas.0401602101 15073337 PMC395939

[42] Zou W, Zhu T, Craft CS, Broekelmann TJ, Mecham RP, Teitelbaum SL. Cytoskeletal dysfunction dominates in DAP12-deficient osteoclasts. J Cell Sci. 2010;123(Pt 17):2955–63. 10.1242/jcs.069872 20720152 PMC2923570

[43] Ishida-Kitagawa N, Tanaka K, Bao X, Kimura T, Miura T, Kitaoka Y, et al Siglec-15 protein regulates formation of functional osteoclasts in concert with DNAX-activating protein of 12 kDa (DAP12). J Biol Chem. 2012;287(21):17493–502. 10.1074/jbc.M111.324194 22451653 PMC3366812

[44] Kameda Y, Takahata M, Komatsu M, Mikuni S, Hatakeyama S, Shimizu T, et al Siglec-15 regulates osteoclast differentiation by modulating RANKL-induced phosphatidylinositol 3-kinase/Akt and Erk pathways in association with signaling Adaptor DAP12. J Bone Miner Res. 2013;28(12):2463–75. 10.1002/jbmr.1989 23677868

[45] Miyamoto K, Ninomiya K, Sonoda KH, Miyauchi Y, Hoshi H, Iwasaki R, et al MCP-1 expressed by osteoclasts stimulates osteoclastogenesis in an autocrine/paracrine manner. Biochemical and biophysical research communications. 2009;383(3):373–7. 10.1016/j.bbrc.2009.04.020 19364494

