# Supplementary figures and images for: Negative feedback loop of bone resorption by NFATc1-dependent induction of Cadm1
Source: PLoS One. 2017 Apr 17;12(4):e0175632. doi: 10.1371/journal.pone.0175632 (PMC5393607; doi:10.1371/journal.pone.0175632)

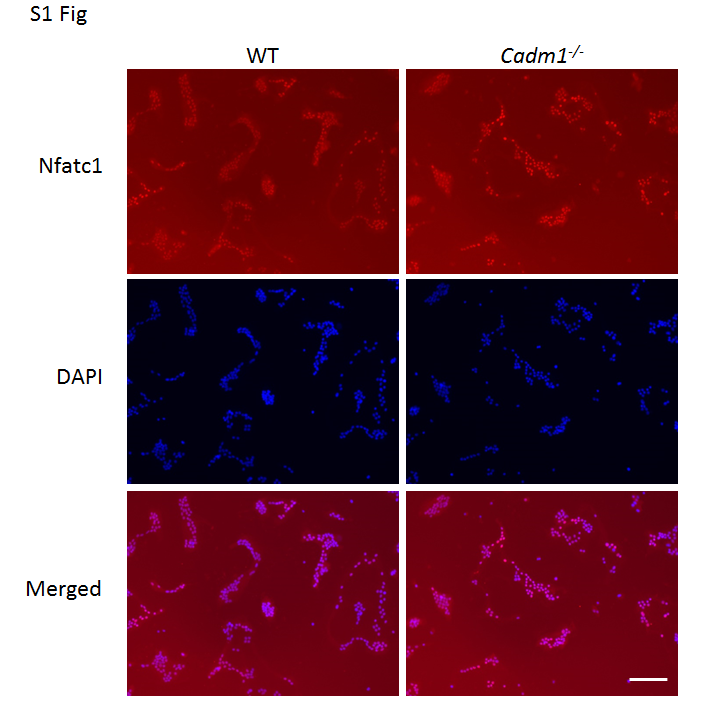

Supplement: S1 Fig — The cells were stained for Nfatc1 using mouse anti-NFATc1 antibody (mouse monoclonal antibody; Santa Cruz Biotechnology, Inc., Dallas, TX, USA) followed by Alexa546-conjugated goat anti-mouse IgG (Invitrogen, Carlsbad, CA, USA). DAPI (Wako Pure Chemicals Industries, Osaka, Japan) was used for a nuclear staining. Nfatc1 was visualized in red, DAPI was double-labeled in blue, and merged images were shown. Bar = 100 μm. (TIF) [file pone.0175632.s001.tif]
